# *trans*-Bis{1-[2-(2,6-diisopropyl­anilino)phenyl]-3-isopropyl­imidazolin-2-ylidenyl-κ*C* ^2^}diiodidopalladium(II) benzene disolvate

**DOI:** 10.1107/S1600536811015431

**Published:** 2011-05-07

**Authors:** Christopher G. Daly, Kuldip Singh, Warren B. Cross

**Affiliations:** a Department of Chemistry, University of Leicester, University Road, Leicester LE1 7RH, England

## Abstract

In the title complex, [PdI_2_(C_24_H_31_N_3_)_2_]·2C_6_H_6_, the Pd^2+^ ion is located on an inversion centre in a slightly distorted square-planar geometry. The angle between the I_2_C_2_ square plane and the mean plane of the *N*-heterocyclic carbene ring is 79.8 (2)°, with I—Pd—C—N torsion angles of −81.1 (6) and −78.2 (5)°. The Pd—carbene and Pd—I distances are 2.016 (6) and 2.5971 (10) Å, respectively.

## Related literature

For a review of *N*-heterocyclic carbenes in late transition metal catalysis, see: Díez-González *et al.* (2009 ▶). For the synthesis of the pro-ligand and crystal structures of related complexes, see: Cross *et al.* (2011 ▶). 
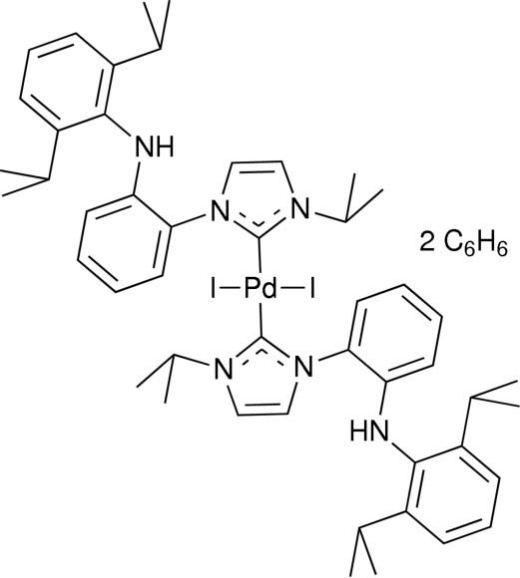


## Experimental

### 

#### Crystal data

[PdI_2_(C_24_H_31_N_3_)_2_]·2C_6_H_6_
*M* *_r_* = 1239.45
Triclinic, 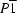

*a* = 8.998 (4) Å
*b* = 12.000 (5) Å
*c* = 13.839 (6) Å
α = 81.572 (8)°
β = 78.819 (9)°
γ = 76.411 (8)°
*V* = 1417.0 (10) Å^3^
*Z* = 1
Mo *K*α radiation
μ = 1.46 mm^−1^
*T* = 150 K
0.16 × 0.11 × 0.06 mm

#### Data collection

Bruker APEX 2000 CCD area-detector diffractometer
Absorption correction: multi-scan (*SADABS*; Bruker, 1998 ▶) *T*_min_ = 0.338, *T*_max_ = 0.831
11207 measured reflections
5500 independent reflections
3317 reflections with *I* > 2σ(*I*)
*R*_int_ = 0.106

#### Refinement

*R*[*F* ^2^ > 2σ(*F* ^2^)] = 0.063
*wR*(*F* ^2^) = 0.128
*S* = 0.88
5500 reflections
319 parameters
H-atom parameters constrained
Δρ_max_ = 0.92 e Å^−3^
Δρ_min_ = −1.27 e Å^−3^

### 

Data collection: *SMART* (Bruker, 2001 ▶); cell refinement: *SAINT* (Bruker, 2000 ▶); data reduction: *SAINT* and *SHELXTL* (Sheldrick, 2008 ▶); program(s) used to solve structure: *SHELXS97* (Sheldrick, 2008 ▶); program(s) used to refine structure: *SHELXL97* (Sheldrick, 2008 ▶); molecular graphics: *SHELXTL*; software used to prepare material for publication: *SHELXTL*.

## Supplementary Material

Crystal structure: contains datablocks I, global. DOI: 10.1107/S1600536811015431/pv2410sup1.cif

Structure factors: contains datablocks I. DOI: 10.1107/S1600536811015431/pv2410Isup2.hkl

Supplementary material file. DOI: 10.1107/S1600536811015431/pv2410Isup3.cdx

Additional supplementary materials:  crystallographic information; 3D view; checkCIF report

## Figures and Tables

**Fig. 1.**
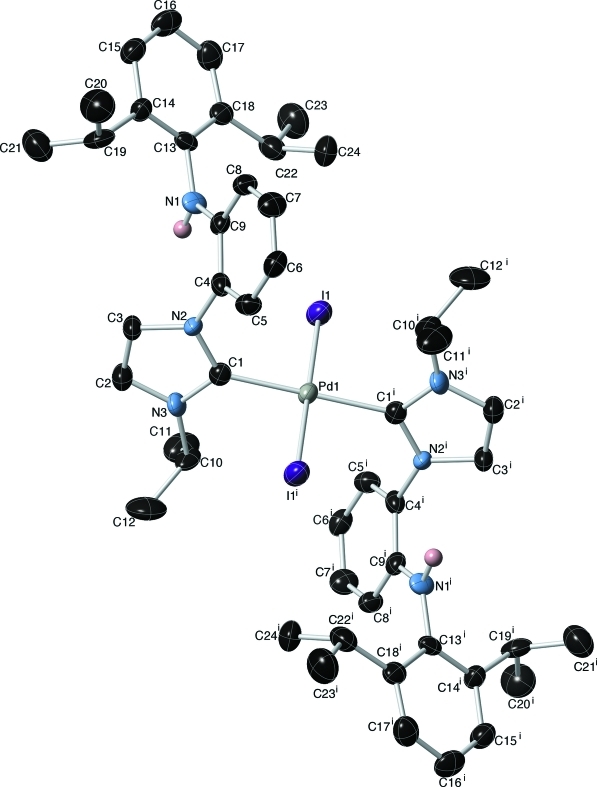
The molecular structure of the title complex, with 50% probablity ellipsoids. Two molecules of benzene and H atoms except N—H have been omitted for clarity. Symmetry operation i = -x + 2, -y, -z.

## supplementary crystallographic information

### Comment

Bifunctional catalysis, in which an electrophilic metal and a nucleophilic ligand act together to activate a substrate, can enable reactions that are not possible using classical catalytic transformations that occur only at the metal centre. We are interested in catalysts in which an *N*-heterocyclic carbene ligand tethers a nucleophilic amido donor to the metal. Previously, we have reported tridentate amido-bis(NHC) (CNC) complexes of palladium and platinum, a bidentate amido-NHC (C,*N*-dipp) complex of palladium and a complex [*trans-*(C_24_H_31_N_3_)PdI_2_(py)], in which the amine-NHC ligand binds to Pd only through the carbene (Cross *et al.*, 2011). In the synthesis of [*trans-*(C_24_H_31_N_3_)PdI_2_(py)], we isolated a single-crystal of the title complex.

The solid state structure of the title complex is shown in Fig. 1. Two NHC and two iodide ligands are coordinated to the square planar Pd, which is located at an inversion centre. The Pd-carbene distance of 2.016 (6) Å is *ca* 0.05 Å longer than in the corresponding complex [*trans-*(C_24_H_31_N_3_)PdI_2_(py)] (Cross *et al.*, 2011), as a consequence of the strong *trans-* influence of the NHC ligand.

### Experimental

The title complex was an unexpected by-product in the synthesis of [*trans-*(C_24_H_31_N_3_)PdI_2_(py)] (Cross *et al.*, 2011). Crystals of the title complex were grown by slow evaporation from benzene.

### Refinement

Hydrogen atoms were included in calculated positions at distances C—H = 0.95 to 1.00 Å and N—H = 0.88 Å in riding mode on the bonded atoms with *U*_iso_(H) set to 1.5 *U*_eq_(C) for methyl H atoms and 1.2 *U*_eq_(C/N) for all other H atoms. The final difference map was essentially featureless with some residual electron density in the close proximity of iodine atom.

### Crystal data

**Table:**

[PdI_2_(C_24_H_31_N_3_)_2_]·2C_6_H_6_	*Z* = 1
*M**_r_* = 1239.45	*F*(000) = 628
Triclinic, *P*1	*D*_x_ = 1.452 Mg m^−^^3^
Hall symbol: -P 1	Mo *K*α radiation, λ = 0.71073 Å
*a* = 8.998 (4) Å	Cell parameters from 580 reflections
*b* = 12.000 (5) Å	θ = 2.4–23.4°
*c* = 13.839 (6) Å	µ = 1.46 mm^−^^1^
α = 81.572 (8)°	*T* = 150 K
β = 78.819 (9)°	Block, yellow
γ = 76.411 (8)°	0.16 × 0.11 × 0.06 mm
*V* = 1417.0 (10) Å^3^	

### Data collection

**Table:**

Bruker APEX 2000 CCD area-detector diffractometer	5500 independent reflections
Radiation source: fine-focus sealed tube	3317 reflections with *I* > 2σ(*I*)
graphite	*R*_int_ = 0.106
φ and ω scans	θ_max_ = 26.0°, θ_min_ = 1.8°
Absorption correction: multi-scan (*SADABS*; Bruker, 1998)	*h* = −11→11
*T*_min_ = 0.338, *T*_max_ = 0.831	*k* = −14→14
11207 measured reflections	*l* = −17→17

### Refinement

**Table:**

Refinement on *F*^2^	Primary atom site location: structure-invariant direct methods
Least-squares matrix: full	Secondary atom site location: difference Fourier map
*R*[*F*^2^ > 2σ(*F*^2^)] = 0.063	Hydrogen site location: inferred from neighbouring sites
*wR*(*F*^2^) = 0.128	H-atom parameters constrained
*S* = 0.88	*w* = 1/[σ^2^(*F*_o_^2^) + (0.035*P*)^2^] where *P* = (*F*_o_^2^ + 2*F*_c_^2^)/3
5500 reflections	(Δ/σ)_max_ = 0.001
319 parameters	Δρ_max_ = 0.92 e Å^−^^3^
0 restraints	Δρ_min_ = −1.27 e Å^−^^3^

### Special details

**Table:**

Geometry. All e.s.d.'s (except the e.s.d. in the dihedral angle between two l.s. planes) are estimated using the full covariance matrix. The cell e.s.d.'s are taken into account individually in the estimation of e.s.d.'s in distances, angles and torsion angles; correlations between e.s.d.'s in cell parameters are only used when they are defined by crystal symmetry. An approximate (isotropic) treatment of cell e.s.d.'s is used for estimating e.s.d.'s involving l.s. planes.
Refinement. Refinement of *F*^2^ against ALL reflections. The weighted *R*-factor *wR* and goodness of fit *S* are based on *F*^2^, conventional *R*-factors *R* are based on *F*, with *F* set to zero for negative *F*^2^. The threshold expression of *F*^2^ > σ(*F*^2^) is used only for calculating *R*-factors(gt) *etc*. and is not relevant to the choice of reflections for refinement. *R*-factors based on *F*^2^ are statistically about twice as large as those based on *F*, and *R*- factors based on ALL data will be even larger.

### Fractional atomic coordinates and isotropic or equivalent isotropic displacement parameters (Å^2^)

**Table:**

	*x*	*y*	*z*	*U*_iso_*/*U*_eq_	
Pd1	1.0000	0.0000	0.0000	0.0245 (2)	
I1	1.08790 (6)	0.17737 (4)	0.03915 (4)	0.03773 (18)	
N1	0.7871 (6)	0.1906 (5)	0.3084 (4)	0.0291 (14)	
H1	0.7552	0.2298	0.2546	0.035*	
N2	0.7258 (6)	0.0747 (5)	0.1601 (4)	0.0245 (13)	
N3	0.6700 (6)	0.1348 (5)	0.0142 (4)	0.0292 (14)	
C1	0.7838 (7)	0.0734 (5)	0.0618 (5)	0.0262 (16)	
C2	0.5403 (8)	0.1765 (6)	0.0799 (5)	0.0382 (19)	
H2	0.4453	0.2227	0.0639	0.046*	
C3	0.5740 (7)	0.1388 (6)	0.1722 (5)	0.0296 (17)	
H3	0.5071	0.1533	0.2332	0.036*	
C4	0.8000 (7)	0.0118 (6)	0.2392 (5)	0.0275 (16)	
C5	0.8455 (8)	−0.1079 (6)	0.2430 (5)	0.0305 (17)	
H5	0.8274	−0.1468	0.1927	0.037*	
C6	0.9170 (8)	−0.1699 (6)	0.3198 (5)	0.0338 (18)	
H6	0.9530	−0.2510	0.3204	0.041*	
C7	0.9359 (8)	−0.1155 (6)	0.3943 (5)	0.0370 (19)	
H7	0.9790	−0.1591	0.4491	0.044*	
C8	0.8928 (8)	0.0033 (6)	0.3907 (5)	0.0319 (17)	
H8	0.9082	0.0398	0.4433	0.038*	
C9	0.8277 (7)	0.0710 (6)	0.3129 (5)	0.0265 (16)	
C10	0.6882 (8)	0.1564 (6)	−0.0949 (5)	0.0354 (19)	
H10	0.7875	0.1056	−0.1231	0.043*	
C11	0.6985 (10)	0.2798 (7)	−0.1303 (6)	0.059 (3)	
H11A	0.5979	0.3307	−0.1104	0.088*	
H11B	0.7263	0.2885	−0.2026	0.088*	
H11C	0.7778	0.3002	−0.1008	0.088*	
C12	0.5553 (11)	0.1221 (9)	−0.1295 (6)	0.071 (3)	
H12A	0.5662	0.0382	−0.1172	0.107*	
H12B	0.5585	0.1460	−0.2006	0.107*	
H12C	0.4561	0.1599	−0.0930	0.107*	
C13	0.7948 (8)	0.2525 (6)	0.3867 (5)	0.0264 (16)	
C14	0.6834 (8)	0.2513 (6)	0.4726 (5)	0.0293 (17)	
C15	0.6914 (8)	0.3156 (6)	0.5452 (5)	0.0370 (19)	
H15	0.6181	0.3148	0.6047	0.044*	
C16	0.8014 (9)	0.3811 (6)	0.5353 (6)	0.043 (2)	
H16	0.8035	0.4250	0.5866	0.052*	
C17	0.9087 (9)	0.3814 (6)	0.4490 (6)	0.041 (2)	
H17	0.9838	0.4273	0.4415	0.049*	
C18	0.9109 (8)	0.3185 (6)	0.3745 (5)	0.0308 (17)	
C19	0.5504 (9)	0.1905 (6)	0.4850 (5)	0.039 (2)	
H19	0.5708	0.1432	0.4282	0.047*	
C20	0.5396 (10)	0.1076 (7)	0.5800 (6)	0.060 (3)	
H20A	0.6394	0.0533	0.5819	0.090*	
H20B	0.4587	0.0649	0.5814	0.090*	
H20C	0.5139	0.1514	0.6376	0.090*	
C21	0.3993 (9)	0.2773 (7)	0.4780 (6)	0.057 (2)	
H21A	0.3738	0.3240	0.5338	0.086*	
H21B	0.3162	0.2366	0.4799	0.086*	
H21C	0.4103	0.3276	0.4156	0.086*	
C22	1.0365 (8)	0.3122 (7)	0.2834 (6)	0.042 (2)	
H22	0.9868	0.3124	0.2247	0.051*	
C23	1.1199 (9)	0.4138 (7)	0.2635 (6)	0.057 (2)	
H23A	1.0434	0.4866	0.2581	0.086*	
H23B	1.1940	0.4070	0.2015	0.086*	
H23C	1.1752	0.4124	0.3182	0.086*	
C24	1.1573 (9)	0.1988 (7)	0.2928 (6)	0.053 (2)	
H24A	1.2033	0.1944	0.3523	0.079*	
H24B	1.2388	0.1961	0.2342	0.079*	
H24C	1.1071	0.1336	0.2978	0.079*	
C25	0.5767 (11)	0.4395 (7)	0.2054 (8)	0.060 (3)	
H25	0.5044	0.3906	0.2232	0.072*	
C26	0.6599 (13)	0.4465 (9)	0.1094 (8)	0.078 (3)	
H26	0.6473	0.4008	0.0623	0.093*	
C27	0.7615 (12)	0.5211 (9)	0.0835 (8)	0.079 (3)	
H27	0.8176	0.5285	0.0181	0.095*	
C28	0.7792 (12)	0.5832 (8)	0.1533 (9)	0.071 (3)	
H28	0.8497	0.6335	0.1365	0.085*	
C29	0.6973 (12)	0.5746 (8)	0.2475 (8)	0.071 (3)	
H29	0.7099	0.6198	0.2950	0.085*	
C30	0.5984 (10)	0.5019 (7)	0.2729 (7)	0.056 (3)	
H30	0.5439	0.4948	0.3388	0.067*	

### Atomic displacement parameters (Å^2^)

**Table:**

	*U*^11^	*U*^22^	*U*^33^	*U*^12^	*U*^13^	*U*^23^
Pd1	0.0217 (4)	0.0279 (4)	0.0209 (4)	−0.0004 (3)	−0.0016 (3)	−0.0040 (3)
I1	0.0378 (3)	0.0405 (3)	0.0365 (3)	−0.0119 (2)	0.0001 (2)	−0.0124 (2)
N1	0.036 (4)	0.024 (3)	0.025 (3)	0.003 (3)	−0.016 (3)	0.003 (3)
N2	0.019 (3)	0.035 (3)	0.018 (3)	−0.002 (3)	−0.002 (2)	−0.008 (3)
N3	0.021 (3)	0.033 (3)	0.030 (4)	0.005 (3)	−0.009 (3)	−0.003 (3)
C1	0.026 (4)	0.025 (4)	0.028 (4)	−0.006 (3)	−0.003 (3)	−0.004 (3)
C2	0.024 (4)	0.049 (5)	0.037 (5)	0.003 (4)	−0.004 (4)	−0.008 (4)
C3	0.019 (4)	0.041 (4)	0.026 (4)	0.000 (3)	−0.002 (3)	−0.003 (3)
C4	0.020 (4)	0.040 (4)	0.020 (4)	−0.007 (3)	−0.002 (3)	0.003 (3)
C5	0.034 (4)	0.035 (4)	0.027 (4)	−0.013 (3)	−0.005 (3)	−0.006 (3)
C6	0.035 (4)	0.029 (4)	0.032 (5)	−0.003 (3)	0.002 (4)	−0.005 (4)
C7	0.044 (5)	0.036 (5)	0.029 (4)	−0.006 (4)	−0.010 (4)	0.003 (4)
C8	0.031 (4)	0.035 (4)	0.029 (4)	0.000 (3)	−0.009 (3)	−0.011 (4)
C9	0.026 (4)	0.029 (4)	0.020 (4)	−0.002 (3)	0.002 (3)	−0.001 (3)
C10	0.040 (5)	0.045 (5)	0.013 (4)	0.006 (4)	−0.005 (3)	0.000 (3)
C11	0.074 (7)	0.061 (6)	0.033 (5)	0.004 (5)	−0.013 (5)	0.001 (4)
C12	0.086 (8)	0.103 (8)	0.033 (5)	−0.019 (6)	−0.024 (5)	−0.015 (5)
C13	0.029 (4)	0.023 (4)	0.029 (4)	−0.002 (3)	−0.015 (3)	−0.005 (3)
C14	0.028 (4)	0.029 (4)	0.032 (4)	−0.003 (3)	−0.002 (3)	−0.011 (3)
C15	0.039 (5)	0.037 (5)	0.032 (5)	−0.003 (4)	0.000 (4)	−0.009 (4)
C16	0.055 (5)	0.027 (4)	0.045 (5)	0.002 (4)	−0.008 (4)	−0.015 (4)
C17	0.035 (5)	0.034 (4)	0.052 (6)	−0.003 (4)	−0.009 (4)	−0.005 (4)
C18	0.032 (4)	0.032 (4)	0.026 (4)	0.002 (3)	−0.011 (3)	−0.002 (3)
C19	0.052 (5)	0.047 (5)	0.020 (4)	−0.015 (4)	−0.002 (4)	−0.012 (4)
C20	0.063 (6)	0.070 (7)	0.047 (6)	−0.026 (5)	−0.003 (5)	0.004 (5)
C21	0.042 (5)	0.075 (7)	0.057 (6)	−0.013 (5)	−0.016 (5)	−0.003 (5)
C22	0.036 (5)	0.062 (6)	0.034 (5)	−0.017 (4)	−0.011 (4)	−0.003 (4)
C23	0.047 (5)	0.065 (6)	0.062 (6)	−0.024 (5)	−0.005 (5)	0.001 (5)
C24	0.031 (5)	0.070 (6)	0.051 (6)	0.004 (4)	−0.001 (4)	−0.020 (5)
C25	0.063 (6)	0.041 (6)	0.069 (7)	0.004 (5)	−0.014 (6)	−0.002 (5)
C26	0.094 (9)	0.064 (7)	0.075 (8)	−0.001 (6)	−0.019 (7)	−0.025 (6)
C27	0.075 (8)	0.077 (8)	0.065 (8)	−0.003 (6)	0.016 (6)	0.000 (6)
C28	0.078 (8)	0.053 (7)	0.082 (8)	−0.023 (6)	−0.019 (7)	0.018 (6)
C29	0.084 (8)	0.050 (6)	0.073 (8)	0.000 (6)	−0.023 (6)	0.002 (6)
C30	0.058 (6)	0.032 (5)	0.062 (7)	0.005 (4)	0.002 (5)	0.002 (5)

### Geometric parameters (Å, °)

**Table:**

Pd1—C1	2.016 (6)	C14—C19	1.513 (9)
Pd1—C1^i^	2.016 (6)	C15—C16	1.377 (9)
Pd1—I1^i^	2.5971 (10)	C15—H15	0.9500
Pd1—I1	2.5971 (10)	C16—C17	1.382 (10)
N1—C9	1.390 (8)	C16—H16	0.9500
N1—C13	1.421 (8)	C17—C18	1.359 (9)
N1—H1	0.8800	C17—H17	0.9500
N2—C1	1.359 (8)	C18—C22	1.518 (9)
N2—C3	1.393 (8)	C19—C21	1.517 (10)
N2—C4	1.421 (8)	C19—C20	1.530 (10)
N3—C1	1.338 (8)	C19—H19	1.0000
N3—C2	1.373 (8)	C20—H20A	0.9800
N3—C10	1.478 (8)	C20—H20B	0.9800
C2—C3	1.359 (9)	C20—H20C	0.9800
C2—H2	0.9500	C21—H21A	0.9800
C3—H3	0.9500	C21—H21B	0.9800
C4—C5	1.395 (9)	C21—H21C	0.9800
C4—C9	1.414 (9)	C22—C24	1.536 (10)
C5—C6	1.380 (9)	C22—C23	1.542 (10)
C5—H5	0.9500	C22—H22	1.0000
C6—C7	1.355 (9)	C23—H23A	0.9800
C6—H6	0.9500	C23—H23B	0.9800
C7—C8	1.382 (9)	C23—H23C	0.9800
C7—H7	0.9500	C24—H24A	0.9800
C8—C9	1.389 (9)	C24—H24B	0.9800
C8—H8	0.9500	C24—H24C	0.9800
C10—C11	1.508 (10)	C25—C30	1.348 (11)
C10—C12	1.531 (10)	C25—C26	1.394 (12)
C10—H10	1.0000	C25—H25	0.9500
C11—H11A	0.9800	C26—C27	1.389 (13)
C11—H11B	0.9800	C26—H26	0.9500
C11—H11C	0.9800	C27—C28	1.355 (13)
C12—H12A	0.9800	C27—H27	0.9500
C12—H12B	0.9800	C28—C29	1.371 (12)
C12—H12C	0.9800	C28—H28	0.9500
C13—C14	1.399 (9)	C29—C30	1.353 (12)
C13—C18	1.425 (9)	C29—H29	0.9500
C14—C15	1.375 (9)	C30—H30	0.9500
			
C1—Pd1—C1^i^	180.0 (5)	C14—C15—C16	122.7 (7)
C1—Pd1—I1^i^	92.78 (18)	C14—C15—H15	118.7
C1^i^—Pd1—I1^i^	87.22 (18)	C16—C15—H15	118.7
C1—Pd1—I1	87.22 (18)	C15—C16—C17	118.5 (7)
C1^i^—Pd1—I1	92.78 (18)	C15—C16—H16	120.7
I1^i^—Pd1—I1	180.00 (2)	C17—C16—H16	120.7
C9—N1—C13	123.0 (6)	C18—C17—C16	122.6 (7)
C9—N1—H1	118.5	C18—C17—H17	118.7
C13—N1—H1	118.5	C16—C17—H17	118.7
C1—N2—C3	109.4 (5)	C17—C18—C13	117.5 (7)
C1—N2—C4	126.2 (5)	C17—C18—C22	122.2 (7)
C3—N2—C4	124.0 (5)	C13—C18—C22	120.2 (6)
C1—N3—C2	111.1 (6)	C14—C19—C21	110.5 (6)
C1—N3—C10	122.9 (6)	C14—C19—C20	112.6 (6)
C2—N3—C10	125.9 (6)	C21—C19—C20	112.0 (7)
N3—C1—N2	106.0 (6)	C14—C19—H19	107.1
N3—C1—Pd1	126.8 (5)	C21—C19—H19	107.1
N2—C1—Pd1	127.1 (5)	C20—C19—H19	107.1
C3—C2—N3	106.8 (6)	C19—C20—H20A	109.5
C3—C2—H2	126.6	C19—C20—H20B	109.5
N3—C2—H2	126.6	H20A—C20—H20B	109.5
C2—C3—N2	106.7 (6)	C19—C20—H20C	109.5
C2—C3—H3	126.7	H20A—C20—H20C	109.5
N2—C3—H3	126.7	H20B—C20—H20C	109.5
C5—C4—C9	120.7 (6)	C19—C21—H21A	109.5
C5—C4—N2	119.4 (6)	C19—C21—H21B	109.5
C9—C4—N2	119.9 (6)	H21A—C21—H21B	109.5
C6—C5—C4	119.9 (7)	C19—C21—H21C	109.5
C6—C5—H5	120.0	H21A—C21—H21C	109.5
C4—C5—H5	120.0	H21B—C21—H21C	109.5
C7—C6—C5	120.2 (7)	C18—C22—C24	110.1 (6)
C7—C6—H6	119.9	C18—C22—C23	113.9 (7)
C5—C6—H6	119.9	C24—C22—C23	108.9 (6)
C6—C7—C8	120.2 (7)	C18—C22—H22	107.9
C6—C7—H7	119.9	C24—C22—H22	107.9
C8—C7—H7	119.9	C23—C22—H22	107.9
C7—C8—C9	122.3 (7)	C22—C23—H23A	109.5
C7—C8—H8	118.9	C22—C23—H23B	109.5
C9—C8—H8	118.9	H23A—C23—H23B	109.5
C8—C9—N1	122.3 (6)	C22—C23—H23C	109.5
C8—C9—C4	116.5 (6)	H23A—C23—H23C	109.5
N1—C9—C4	121.3 (6)	H23B—C23—H23C	109.5
N3—C10—C11	111.4 (6)	C22—C24—H24A	109.5
N3—C10—C12	109.1 (6)	C22—C24—H24B	109.5
C11—C10—C12	112.4 (7)	H24A—C24—H24B	109.5
N3—C10—H10	107.9	C22—C24—H24C	109.5
C11—C10—H10	107.9	H24A—C24—H24C	109.5
C12—C10—H10	107.9	H24B—C24—H24C	109.5
C10—C11—H11A	109.5	C30—C25—C26	120.2 (10)
C10—C11—H11B	109.5	C30—C25—H25	119.9
H11A—C11—H11B	109.5	C26—C25—H25	119.9
C10—C11—H11C	109.5	C27—C26—C25	119.2 (10)
H11A—C11—H11C	109.5	C27—C26—H26	120.4
H11B—C11—H11C	109.5	C25—C26—H26	120.4
C10—C12—H12A	109.5	C28—C27—C26	118.8 (10)
C10—C12—H12B	109.5	C28—C27—H27	120.6
H12A—C12—H12B	109.5	C26—C27—H27	120.6
C10—C12—H12C	109.5	C27—C28—C29	121.2 (10)
H12A—C12—H12C	109.5	C27—C28—H28	119.4
H12B—C12—H12C	109.5	C29—C28—H28	119.4
C14—C13—N1	119.1 (6)	C30—C29—C28	120.1 (10)
C14—C13—C18	121.4 (6)	C30—C29—H29	120.0
N1—C13—C18	119.5 (6)	C28—C29—H29	120.0
C15—C14—C13	117.3 (6)	C25—C30—C29	120.4 (9)
C15—C14—C19	119.5 (6)	C25—C30—H30	119.8
C13—C14—C19	123.0 (6)	C29—C30—H30	119.8
			
C2—N3—C1—N2	0.5 (8)	C1—N3—C10—C11	−107.3 (8)
C10—N3—C1—N2	178.5 (6)	C2—N3—C10—C11	70.3 (9)
C2—N3—C1—Pd1	−177.1 (5)	C1—N3—C10—C12	128.0 (7)
C10—N3—C1—Pd1	0.9 (9)	C2—N3—C10—C12	−54.3 (9)
C3—N2—C1—N3	−0.4 (7)	C9—N1—C13—C14	−73.6 (8)
C4—N2—C1—N3	173.8 (6)	C9—N1—C13—C18	109.2 (7)
C3—N2—C1—Pd1	177.2 (5)	N1—C13—C14—C15	−177.7 (6)
C4—N2—C1—Pd1	−8.6 (9)	C18—C13—C14—C15	−0.6 (10)
I1^i^—Pd1—C1—N3	−81.1 (6)	N1—C13—C14—C19	−2.4 (10)
I1—Pd1—C1—N3	98.9 (6)	C18—C13—C14—C19	174.7 (7)
I1^i^—Pd1—C1—N2	101.8 (5)	C13—C14—C15—C16	1.1 (11)
I1—Pd1—C1—N2	−78.2 (5)	C19—C14—C15—C16	−174.3 (7)
C1—N3—C2—C3	−0.5 (8)	C14—C15—C16—C17	−0.5 (12)
C10—N3—C2—C3	−178.4 (6)	C15—C16—C17—C18	−0.9 (12)
N3—C2—C3—N2	0.3 (8)	C16—C17—C18—C13	1.4 (11)
C1—N2—C3—C2	0.0 (8)	C16—C17—C18—C22	−175.2 (7)
C4—N2—C3—C2	−174.2 (6)	C14—C13—C18—C17	−0.6 (10)
C1—N2—C4—C5	−57.2 (9)	N1—C13—C18—C17	176.4 (6)
C3—N2—C4—C5	116.1 (7)	C14—C13—C18—C22	176.0 (7)
C1—N2—C4—C9	122.4 (7)	N1—C13—C18—C22	−6.9 (10)
C3—N2—C4—C9	−64.3 (8)	C15—C14—C19—C21	68.8 (9)
C9—C4—C5—C6	0.7 (10)	C13—C14—C19—C21	−106.4 (8)
N2—C4—C5—C6	−179.7 (6)	C15—C14—C19—C20	−57.4 (9)
C4—C5—C6—C7	3.4 (10)	C13—C14—C19—C20	127.4 (7)
C5—C6—C7—C8	−4.1 (10)	C17—C18—C22—C24	100.7 (8)
C6—C7—C8—C9	0.7 (11)	C13—C18—C22—C24	−75.9 (8)
C7—C8—C9—N1	−178.7 (6)	C17—C18—C22—C23	−22.0 (10)
C7—C8—C9—C4	3.2 (10)	C13—C18—C22—C23	161.5 (7)
C13—N1—C9—C8	−6.2 (10)	C30—C25—C26—C27	−2.0 (14)
C13—N1—C9—C4	171.8 (6)	C25—C26—C27—C28	1.4 (16)
C5—C4—C9—C8	−3.9 (9)	C26—C27—C28—C29	−1.0 (16)
N2—C4—C9—C8	176.5 (6)	C27—C28—C29—C30	1.1 (15)
C5—C4—C9—N1	178.0 (6)	C26—C25—C30—C29	2.1 (14)
N2—C4—C9—N1	−1.6 (9)	C28—C29—C30—C25	−1.6 (14)

Symmetry codes: (i) −*x*+2, −*y*, −*z*.
